# Surface-Enhanced Raman Spectroscopy for Adenine Detection in Five Selected Bacterial Strains Under Stress Conditions

**DOI:** 10.3390/s25154629

**Published:** 2025-07-26

**Authors:** Mona Ghazalová, Pavlína Modlitbová, Ota Samek, Katarína Rebrošová, Martin Šiler, Jan Ježek, Zdeněk Pilát

**Affiliations:** 1 Institute of Scientific Instruments of the Czech Academy of Sciences, v.v.i., Královopolská 147, 612 00 Brno, Czech Republic; 2 Department of Microbiology, Faculty of Medicine of Masaryk University and St. Anne’s, University Hospital, Pekařská 53, 656 91 Brno, Czech Republic

**Keywords:** SERS, Ag-NPs, Au-NPs, phenylalanine, *Escherichia coli*, *Staphylococcus aureus*

## Abstract

**Highlights:**

**What are the main findings?**

- Adenine was detected as a stress marker in five bacterial strains.
- Stable Au and Ag NPs enabled SERS-based analysis of secreted bacterial metabolites.

**What is the implication of the main findings?**

- Label-free SERS detects metabolic stress responses in bacteria cost-effectively.
- Gold NPs enable long-term, reproducible Raman-based bacterial stress detection.

**Abstract:**

This pilot study investigated the metabolic responses of five selected bacteria to physiological stress. Surface-enhanced Raman spectroscopy was used to analyze spectral changes associated with the release of adenine, a key metabolite indicative of stress conditions. Laboratory-synthesized spherical silver and gold nanoparticles, which remained stable over an extended period, were employed as enhanced surfaces. Bacterial cultures were analyzed under standard conditions and in the presence of a selected stressor—demineralized water—inducing osmotic stress. The results showed that the adenine signal originated from metabolites released into the surrounding environment rather than directly from the bacterial cell wall. The study confirms the suitability of these cost-effective and easily synthesized stable nanoparticles for the qualitative detection of bacterial metabolites using a commercially available Raman instrument.

## 1. Introduction

Raman spectroscopy (RS) is a technique that uses lasers to obtain information about molecular vibrations within a sample. When the laser interacts with molecules, it partly undergoes inelastic Raman scattering which reflects the sample’s chemical composition [1]. This spectrum serves as a molecular fingerprint and can be used to identify bacteria based on their unique biochemical profiles. Beyond bacterial identification, RS also allows the detection of various cellular components and metabolites, such as proteins, lipids, nucleic acids, and small molecules like adenine or phenylalanine [2]. However, a common limitation of standard RS is the inherently weak signal, which often makes it difficult to achieve the sensitivity and specificity needed for reliable bacterial discrimination [3]. This challenge has led to the development of signal-enhancement techniques, such as laser tweezers Raman spectroscopy (LTRS) [4] and surface-enhanced Raman spectroscopy (SERS) [5,6], which significantly boost the Raman signal and allow for detection of even trace amounts of analytes.

Surface-enhanced Raman spectroscopy offers a significant enhancement of the Raman signal by placing the studied sample in the proximity of plasmonic nanostructures, enabling more accurate identification of bacteria. In principle, SERS enhances the detection sensitivity up to the single molecule level [7,8,9]. Therefore, SERS is a promising technique for the rapid detection of bacteria as well as biomarkers associated with certain types of cancer and other diseases [5,6]. It is crucial to improve our understanding of bacterial response to different environmental stresses. One way to detect the stress response of bacteria is by analyzing their unique Raman or SERS spectra and the metabolites they release under specific environmental conditions. In this work, we take advantage of adenine and similar metabolites, which can be detected in the bacterial cell [10] and/or released into the environment as a signaling molecule [11,12].

Adenosine and its derivatives, such as ATP (adenosine triphosphate), are molecules that serve as a source of chemical energy, a building block of nucleic acids, and a relevant messaging/signaling molecule [12]. Since bacteria do not have mitochondria, the ATPase and electron transport chain are located in the cytoplasmic membrane [10,13]. The membrane is responsible for many vital processes from energy conversion and nutrition processing to molecular synthesis [13]. SERS has been shown to be effective in recognizing and describing various bacterial species as well as improving understanding of chemically driven metabolic changes [10]. There are several approaches to obtaining the SERS spectra [10] of bacteria: bacteria can be placed directly on the SERS substrate [14,15], colloidal silver can be formed on or inside individual bacteria [9], and, finally, bacteria and NPs (nanoparticles) can be mixed together and placed on a flat surface [11,16]. With the utilization of SERS, we can identify purine compounds, e.g., adenine-related compounds, on the bacterial cell wall. Although primarily located within the bacterial cell, some purine residues appear on the inner surface of the cell wall, making them detectable by SERS [17]. For example, SERS has been used to detect adenine released by *E. coli* cells exposed to starvation, where increased levels of extracellular adenine indicated metabolic shifts associated with stress adaptation [14]. Similarly, adenine has been identified as a dominant SERS marker in the analysis of bacterial lysates and culture extracts, reflecting both intracellular release and nucleic acid breakdown [18]. These findings highlight the potential of SERS-based detection of adenine as a non-invasive, rapid approach to probe microbial stress responses or to assess metabolic activity in complex biological samples. This detection not only aids in understanding bacterial structure but also provides insights into the metabolic activities and potential vulnerabilities of the bacteria.

This study aims to explore the practical applicability of SERS for stress biomarker detection in bacterial systems using cost-effective and easily synthesized spherical NPs. By applying both silver and gold colloids synthesized via a modified Lee–Meisel protocol [19], we demonstrate their long-term stability and feasibility for detecting adenine as a stress-associated metabolite. Rather than optimizing enhancement per se, we focus on evaluating the suitability and reproducibility of these NPs for microbial metabolite detection under physiological stress, potentially contributing to future point-of-care (POC) screening applications.

## 2. Experimental Section

### 2.1. Materials and Reagents

Silver nitrate (AgNO_3_, pure) and L-ascorbic acid (LL, pure) used for nanoparticle synthesis were obtained from Penta (Chrudim, Czech Republic). Chloroauric acid trihydrate (HAuCl_4_·3H_2_O) was purchased from Sigma-Aldrich s.r.o. (St. Louis, MO, USA). Agar-Agar was sourced from Carl Roth (Karlsruhe, Germany), and Luria–Bertani (LB) broth was purchased from Sigma-Aldrich s.r.o. (St. Louis, MO, USA).

### 2.2. Synthesis of Nanoparticles

**Figure 1:**
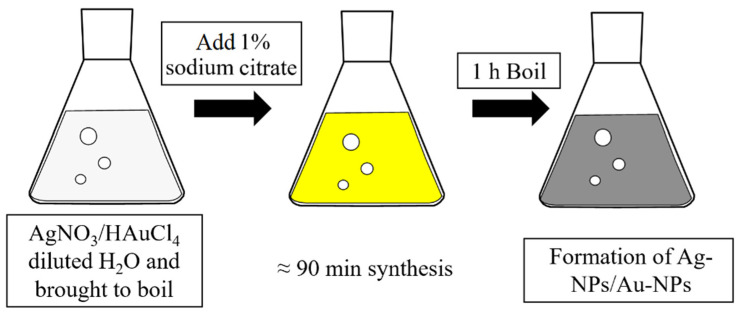
Schematic representation of the NP synthesis process.

The Ag-NPs and Au-NPs were synthesized using the method by Lee and Meisel [19]. To obtain the Ag- and Au-NPs, 18 mg of AgNO_3_/240 mg of HAuCl_4_ were diluted in 100 mL/500 mL of dH_2_O and brought to boil. A solution of 2 mL/50 mL 1% sodium citrate was then added to the AgNO_3_/HAuCl_4_ solution. The solution was mixed while stirring at a temperature of 95 °C for 1 h. After 1 h, the solution was left to cool down while stirring. A magnetic stir bar was used for continuous stirring. The schematic representation of the synthesis process is shown in Figure 1.

### 2.3. Characterization of Nanoparticles

To further characterize the NPs, it was important to visualize their size and morphology. A MAGELLAN 400 scanning electron microscope (ThermoFisher, Brno, Czech Republic) was utilized. The prepared NPs were centrifuged for 2 min at 3287× *g*. Then, 3 µL of NPs were sprayed onto a copper mesh with a formvar layer which was held with crossed tweezers in a laminar flow box until the solvent evaporated. Then, the size distribution and zeta potential of the NPs (directly in their aquatic dispersions) were measured with a ZetaSizer Nano ZS (Malvern Panalytical, Worcestershire, UK) at the Brno University of Technology, Faculty of Chemistry (Brno, Czech Republic). An average of 5 measurements of zeta potential were taken, as well as 3 measurements of the size distribution.

### 2.4. Microorganisms

The bacteria *Escherichia coli* K-12 was received from Prof. Ute Neugebauer, University Clinic, in Jena, Germany. The bacteria *Staphylococcus aureus* CCM4890, *Staphylococcus epidermis* CCM 4418, *Enterococcus faecalis* CCM 4224, and *Staphylococcus lugdunensis* CCM 4069 were obtained from Czech Collection of Microorganisms, Brno, Czech Republic.

### 2.5. Microbiology

In order to obtain bacteria for SERS-based analysis, agar plates with Luria–Bertani (LB) agar (10 g Tryptone, 5 g yeast extract, 5 g NaCl, 2% agar, suspended in 1 L DI (deionized) water and autoclaved at 121 °C for 15 min) were inoculated from stock culture kept at −80 °C, which was prepared by mixing 0.5 mL of LB broth (10 g Tryptone, 5 g yeast extract, 5 g NaCl, suspended in 1 L DI water and autoclaved at 121 °C for 15 min) with bacteria and 0.5 mL of sterile 50% glycerol. The inoculated plates were incubated at 37 °C for 24 h. All the bacteria in the experiments were in the late exponential growth phase.

**Figure 2:**
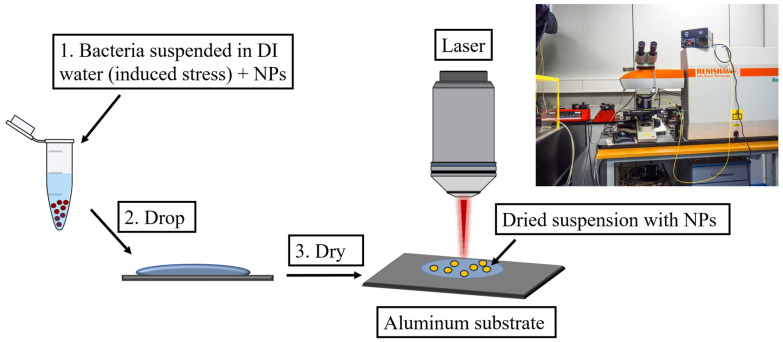
SERS measurement approach. 1. Mix the spent medium or bacterial suspension with NPs in 1:1 ratio. 2. Deposit 1 μL of the mixture on Al foil. 3. Allow to dry. Followed by SERS measurement.

After the incubation, one colony of each culture was collected using a 10 µL inoculation loop, washed twice with 1 mL of DI water to remove the growth medium, and resuspended in 1 mL of DI water. After each washing step, the bacteria were separated by centrifugation at 6708× *g* for 5 min in an Eppendorf Minispin centrifuge (Eppendorf, Hamburg, Germany). The control variant used LB broth instead of DI water during the washing step. This allowed us to observe the bacterial behavior both with and without osmotic stress. Subsequently, 25 μL of the bacteria and 25 μL of the Ag- or Au-NPs were mixed in a 1.5 mL Eppendorf tube. The mixtures were deposited onto an Al foil surface and allowed to dry for approximately 10 min for subsequent analysis. The schematic experimental setup is illustrated in Figure 2. Moreover, *S. aureus* and *E. coli* were also measured by conventional Raman spectroscopy after harvesting the cells from the growth medium and smearing them on an Al foil without any prior treatment.

### 2.6. Raman Spectroscopy and Surface-Enhanced Raman Spectroscopy

Raman and SERS spectra were acquired using a Renishaw InVia Raman spectrometer (Renishaw, Wotton-under-Edge, UK) equipped with a 785 nm excitation laser and a 20× microscope objective (N-PLAN EPI 0.4, Leica Microsystems, Wetzlar, Germany). The system uses a confocal microscope setup with precise spatial resolution and a CCD detector optimized for low-light conditions [20]. For each sample, 10 spectra were recorded from different points along the perimeter of the dried droplet (“coffee ring” region) and subsequently averaged to improve reproducibility. The integration time for each spectrum was 10 s. This configuration enables efficient and spatially resolved analysis of analytes adsorbed on the nanoparticle surface.

### 2.7. Data Analysis

The Raman or SERS spectra were averaged from 10 measurements per sample. For data processing and analysis, we used MATLAB R2024a-based Raman data-processing software “Raman2”, developed at ISI CAS. The fluorescence background was removed by the rolling-circle spectral filter, which was proposed in the work by Brandt et al. [21]. The background was removed with the following parameters: circle diameter 1000 pixels (approx. 1000 cm^−1^), 10 passes, and 300 pixels (approx. 300 cm^−1^), 30 passes. The removal of high-frequency noise was performed with a Savitzky–Golay filter [22] (2 passes, order 2 and frame length 7 pixels). This increased the signal–noise ratio of the spectra.

## 3. Results and Discussion

### 3.1. Properties of the Nanoparticles

**Figure 3:**
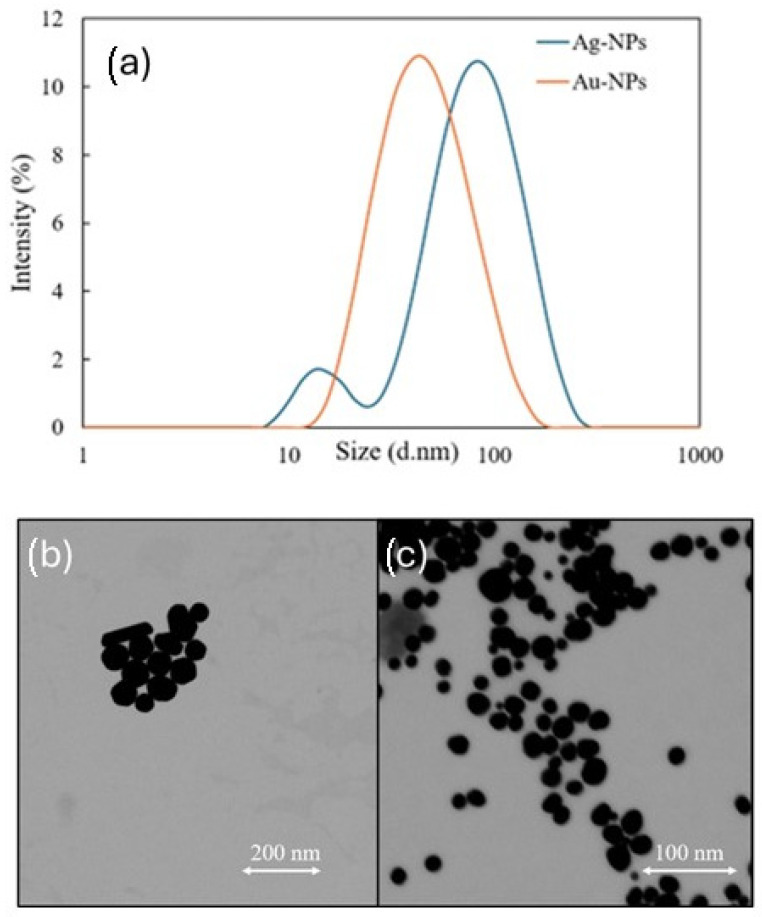
(**a**) Size distribution of Au-NPs and Ag-NPs 6 months after synthesis. The DLS measurements showing the size distribution. (**b**) STEM image of synthesized Ag-NPs and (**c**) STEM image of synthesized Au-NPs. The scale bars are shown directly in the figures.

The size distribution of the prepared NPs was estimated with a ZetaSizer. The mean diameter of the Au-NPs was 34.9 ± 0.11 nm, and for Ag-NPs, it was 60.3 ± 0.13 nm. However, the size distribution of the Ag-NPs revealed two distinct peaks: a primary peak in the 50–100 nm range and a secondary peak around 16 nm (see Figure 3). The polydispersity index values were 0.237 ± 0.00 and 0.294 ± 0.03, respectively, suggesting a relatively uniform size distribution for both NPs, although the Ag-NPs exhibited slightly higher variability. The dynamic light scattering (DLS) measurements were consistent with the scanning transmission electron microscopy (STEM) results, which confirm the uniformity of Au-NPs while revealing larger sizes and varied shapes for Ag-NPs (see Figure 3). To assess colloidal stability over time, zeta potential measurements were conducted six months after their synthesis. The zeta potential of the NPs was analyzed, with average values of −37.56 mV for Au-NPs and −21.76 mV for Ag-NPs, implying better colloidal stability for Au-NPs as no considerable clustering was detected in the indicated time frame.

### 3.2. Raman Spectroscopy

**Figure 4:**
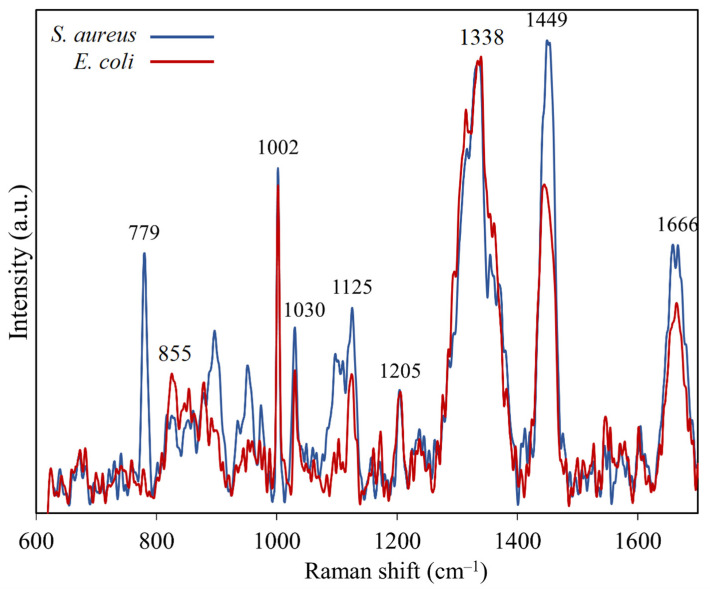
Raman spectrum of bacterium *S. aureus* (blue) and *E. coli* (red) without NPs, averaged from 10 spectra per sample. The spectra were normalized to the phenylalanine peak at 1002 cm^−1^.

Firstly, the cells of *E. coli* and *S. aureus* were spectrographed using standard RS, and the spectra were normalized to the area of the phenylalanine peak at 1002 cm^−1^. The Raman spectra are shown in Figure 4. The Raman peaks and the molecules tentatively assigned to them are shown in Table 1 for reference. The detected Raman signals in *S. aureus* belonged to molecules typically found in bacterial cells. *E. coli* also shows Raman peaks of typical bacterial constituents, but with a notable absence of the peak at 779 cm^−1^. Instead, a peak at 855 cm^−1^ is present, which corresponds to tyrosine. The Raman peak assignments for *S. aureus* and *E. coli* bacteria were based on the article by De Gelder et al. [23]. The presented spectra chiefly serve to demonstrate that the adenine signal at 730 cm^−1^ was not dominant in the Raman spectra obtained with conventional Raman spectroscopy.

### 3.3. Surface-Enhanced Raman Spectroscopy for Detection of Adenine in Bacterial Cells

**Figure 5:**
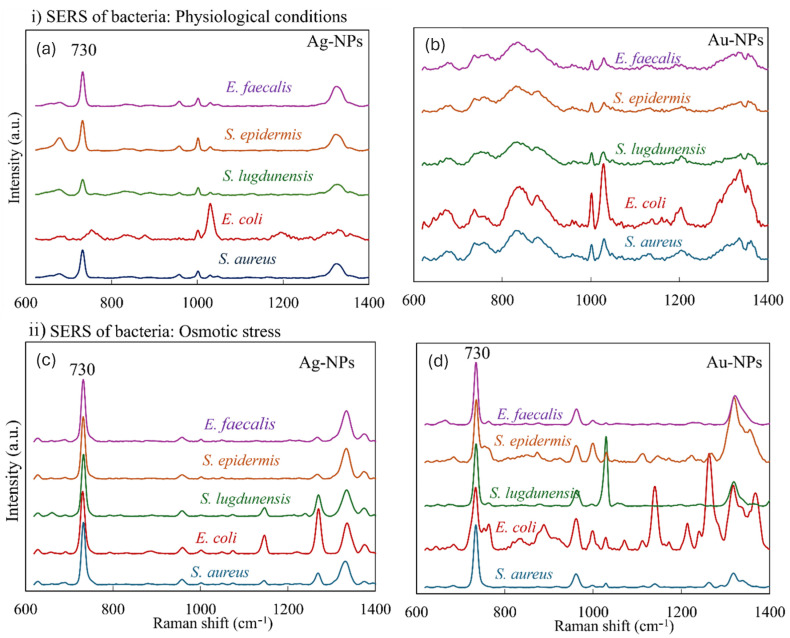
(**i**) The SERS spectra of bacteria with NPs in physiological conditions. The figure presents SERS spectra of bacterial cells with Au-NPs and Ag-NPs. (**a**) Bacterial suspension with Ag-NPs; (**b**) bacterial suspension with Au-NPs. (**ii**) The SERS spectra of bacteria with NPs in osmotic stress caused by DI water. (**c**) Bacterial suspension with Ag-NPs; (**d**) bacterial suspension with Au-NPs. A dominant peak of adenine was observed at 730 cm^−1^. Averaged from 10 spectra per sample. The spectra were normalized to the adenine peak at 730 cm^−1^.

To investigate bacterial metabolism under non-stressful conditions, we conducted SERS analysis of intact bacterial cells water (Figure 5a,b). Under such physiological conditions, only minimal release of adenine-related compounds is expected. Nevertheless, a distinct SERS peak at ~730 cm^−1^ was still detectable in samples measured with Ag-NPs. This observation aligns with the well-known high enhancement efficiency of silver nanoparticles in SERS [24], but it also suggests that Ag-NPs are capable of detecting even trace amounts of adenine naturally present in non-stressed cells. While Ag-NPs yielded high SERS intensity and overall signal enhancement, their spectral profiles did not allow us to reliably distinguish between stressed and non-stressed bacterial states under our experimental conditions. This observation is not meant to generalize about Ag-NPs’ performance in all contexts, but rather to reflect our specific findings, where Au-NPs yielded spectra with better consistency in identifying adenine as a stress-associated metabolite.

In contrast, SERS measurements using Au-NPs did not show a detectable adenine peak under the same non-stressful conditions. In our experimental setup, Au-NPs yielded observable adenine signals specifically under osmotic stress conditions, which suggests their potential utility for selectively identifying stress-induced adenine release. Further mechanistic investigations are needed to fully elucidate the basis of this selectivity. For instance, *E. coli* did not produce a significant adenine peak when exposed to Au- or Ag-NPs in physiological conditions (Figure 5a,b), yet a clear adenine signal appeared after osmotic stress induction using DI water (Figure 5c,d). This confirms that the dominant 730 cm^−1^ peak observed under stress conditions arises from compounds released into the extracellular environment, indicating adenine as a biomarker of bacterial stress detectable by citrate-coated Au-NPs under our experimental conditions. The citrate-coated Au-NPs thus serve as a more straightforward and reliable SERS probe for detecting adenine as a biomarker of bacterial stress. The specific interaction of *E. coli* K-12 with citrate-coated nanoparticles is further discussed in [25]. Our analysis is therefore qualitative: once spectra are normalized to the adenine band, the key question becomes whether that band is detectable at all. Table 2 already provides this binary outcome for each strain and substrate, which is sufficient for rapid stress screening and avoids potentially misleading semi-quantitative comparisons.

The SERS signal observed at ~730 cm^−1^ is typically attributed to adenine or closely related purine derivatives [9,10,17,26]. Given the short effective range of the SERS effect (tens of nanometers), it is unlikely that these signals originate from intracellular components, which are shielded from the nanoparticle surface [27]. Efrima and Zeiri [9] discussed in their review that the spectra of the bacterial cell wall contain prominent signals of certain biomolecules, e.g., Flavin Adenine Nucleotide (FAD), with the peaks at 735 cm^−1^ and 1330 cm^−1^ associated with partially or fully reduced FAD [9,17,26] or with other adenine-containing molecules (e.g., ATP) [8,9,10,28,29]. Kubryk et al. [30] clarified the origin of the SERS peak at 730 cm^−1^ as adenine using stable isotopes of ^13^C and ^15^N, specifically the in-plane ring breathing mode of adenine. A variety of other adenine-containing molecules and products of the purine breakdown pathway may be responsible for this peak [30].

Because the adenine peak is either present or absent after normalization, we interpret its detectability, not its absolute amplitude, as the decisive indicator of stress (see Table 2). This table clearly demonstrated that standard RS fails to detect adenine under our experimental conditions. In contrast, SERS detected an intense adenine peak within seconds, with most intensive signal obtained in the presence of Ag-NPs.

In practical terms, Ag-NPs maximize sensitivity, registering a YES signal even in non-stressed cells, whereas Au-NPs register the same YES only after osmotic stress. The complementary behavior of the two colloids empowers users to tune the assay either for broad metabolite surveillance (Ag) or for stress-specific detection (Au).

## 4. Conclusions and Future Prospects

This pilot study demonstrates the feasibility of using simply synthesized, long-term stable NPs for label-free detection of bacterial metabolites. By comparing Raman and SERS spectra of bacterial samples under osmotic stress, we showed that SERS enables the detection of stress-induced biomarkers, most notably adenine, undetectable by conventional Raman spectroscopy. A characteristic peak at 730 cm^−1^ confirmed the presence of adenine in both bacterial suspensions and their surrounding medium, indicating active metabolite release under stress.

Critically, we found that Au-NPs enable selective detection of adenine only under stress conditions, whereas Ag-NPs also detect trace amounts of adenine present under non-stress conditions. Our experimental findings demonstrate that both Ag- and Au-NPs enable sensitive detection of adenine released under bacterial stress conditions. Au-NPs uniquely exhibited adenine detection predominantly under stress conditions within our specific experimental framework, suggesting potential applicability in distinguishing stress states. Future research, including mechanistic and affinity studies, is necessary to establish broader conclusions on selectivity and the utility of these nanoparticles in clinical and diagnostic contexts.

Physiological stress, including that induced during sample handling, such as brief exposure to DI water, can lead to the release of adenine-related metabolites, signaling cellular energy deficiency [5]. In response, bacteria may suspend growth and division as part of a survival strategy, conserving resources until environmental conditions improve [14]. The accumulation of such metabolites may thus represent a preparatory phase for future recovery [31]. Monitoring the surrounding medium (spent medium) enables direct access to these soluble metabolic markers without interference from cellular components, simplifying SERS signal attribution and improving identification accuracy.

In routine clinical diagnostics, bacterial identification still heavily relies on cultivation, followed by techniques such as MALDI-TOF MS (matrix-assisted laser desorption/ionization–time-of-flight mass spectrometry) or biochemical tests on selective media. While MALDI offers faster identification post-cultivation, it requires costly instrumentation and is limited in throughput. In more complex or non-cultivable cases, molecular methods such as PCR (polymerase chain reaction) or ELISA (enzyme-linked immunosorbent assay) may be used, but their widespread application is restricted by technical demands, cost, and the need for trained personnel [5,9,32,33]. Taken together, the qualitative YES/NO read-out embodied in Table 2 demonstrates that substrate choice alone can switch the assay from maximum sensitivity (Ag) to stress selectivity (Au). These limitations underscore the need for simpler, faster, and more accessible analytical tools capable of detecting bacteria or their metabolic products with minimal sample preparation [8,34].

SERS, combined with tailored NP substrates, offers a promising path forward. The simplicity of NP synthesis, coupled with their sensitivity and selectivity, supports the development of portable, POC (point-of-care) diagnostic tools. Future work should focus on optimizing NP formulations and SERS substrates to improve reproducibility, as well as expanding the technique to a broader range of bacterial species and stress conditions. Ultimately, integrating SERS into clinical workflows could streamline bacterial identification and accelerate antibiotic susceptibility testing, improving patient outcomes and supporting antimicrobial stewardship efforts.

## Figures and Tables

**Table 1:** Band assignments [23] in the Raman spectra of *S. aureus* and *E. coli.*

*S. aureus*		*E. coli*	
Raman Shift (cm^−1^)	Tentative Assignment	Raman Shift (cm^−1^)	Tentative Assignment
779	Cytosine, uracil	855	Tyrosine
1002	Phenylalanine	1002	Phenylalanine
1030	Phenylalanine, protein side chains	1030	Phenylalanine, protein side chains
1125	Lipids	1125	Lipids
1205	Phenylalanine, tyrosine, tryptophane	1205	Phenylalanine, tyrosine, tryptophane
1338	Protein side chains	1338	Protein side chains
1449	Protein side chains, lipids	1449	Protein side chains, lipids
1666	Lipids	1666	Lipids

**Table 2:** RS versus SERS for detection of adenine in bacteria in osmotic stress and physiological “unstressed” conditions. Red cross means—not detectable, green cross means—detectable.

	Bacterial Strain	Adenine Detectability		
		Raman	SERS Ag-NPs	SERS Au-NPs
OSMOTIC STRESS	*E. coli*	**✗**	**✔**	**✔**
	*S. aureus*	**✗**	**✔**	**✔**
	*S. lugdunensis*	**✗**	**✔**	**✔**
	*S. epidermis*	**✗**	**✔**	**✔**
	*E. faecalis*	**✗**	**✔**	**✔**
PHYSIOLOGICAL “UNSTRESSED” CONDITIONS	*E. coli*	**✗**	**✗**	**✗**
	*S. aureus*	**✗**	**✔**	**✗**
	*S. lugdunensis*	**✗**	**✔**	**✗**
	*S. epidermis*	**✗**	**✔**	**✗**
	*E. faecalis*	**✗**	**✔**	**✗**

## Data Availability

To access the Raman spectral data used in this publication, go to Raman Base [35], https://ramanbase.org (accessed on 20 July 2025) and follow the instructions.

## Abbreviations

The following abbreviations are used in this manuscript:

ATP	Adenosine Triphosphate
DI	Deionized
DLS	Dynamic Light Scattering
ELISA	Enzyme-Linked Immunosorbent Assay
LB	Luria–Bertani
LTRS	Laser Tweezers Raman Spectroscopy
MALDI-TOF MS	Matrix-Assisted Laser Desorption/Ionization–Time-of-Flight Mass Spectrometry
NP	Nanoparticles
PCR	Polymerase Chain Reaction
POC	Point of Care
RS	Raman Spectroscopy
SERS	Surface-Enhanced Raman Spectroscopy
STEM	Scanning Transmission Electron Microscopy
